# Deep Learning: A Review for the Radiation Oncologist

**DOI:** 10.3389/fonc.2019.00977

**Published:** 2019-10-01

**Authors:** Luca Boldrini, Jean-Emmanuel Bibault, Carlotta Masciocchi, Yanting Shen, Martin-Immanuel Bittner

**Affiliations:** ^1^Dipartimento di Diagnostica per Immagini, Radioterapia Oncologica ed Ematologia, Università Cattolica del Sacro Cuore, Rome, Italy; ^2^Radiation Oncology Department, Georges Pompidou European Hospital, Assistance Publique—Hôpitaux de Paris, Paris Descartes University, Paris Sorbonne Cité, Paris, France; ^3^Department of Engineering Science, University of Oxford, Oxford, United Kingdom; ^4^CRUK/MRC Oxford Institute for Radiation Oncology, University of Oxford, Oxford, United Kingdom

**Keywords:** machine learning, deep learning, modeling, radiation oncology, clinical oncology

## Abstract

**Introduction:** Deep Learning (DL) is a machine learning technique that uses deep neural networks to create a model. The application areas of deep learning in radiation oncology include image segmentation and detection, image phenotyping, and radiomic signature discovery, clinical outcome prediction, image dose quantification, dose-response modeling, radiation adaptation, and image generation. In this review, we explain the methods used in DL and perform a literature review using the Medline database to identify studies using deep learning in radiation oncology. The search was conducted in April 2018, and identified studies published between 1997 and 2018, strongly skewed toward 2015 and later.

**Methods:** A literature review was performed using PubMed/Medline in order to identify important recent publications to be synthesized into a review of the current status of Deep Learning in radiation oncology, directed at a clinically-oriented reader. The search strategy included the search terms “radiotherapy” and “deep learning.” In addition, reference lists of selected articles were hand searched for further potential hits of relevance to this review. The search was conducted in April 2018, and identified studies published between 1997 and 2018, strongly skewed toward 2015 and later.

**Results:** Studies using DL for image segmentation were identified in Brain (*n* = 2), Head and Neck (*n* = 3), Lung (*n* = 6), Abdominal (*n* = 2), and Pelvic (*n* = 6) cancers. Use of Deep Learning has also been reported for outcome prediction, such as toxicity modeling (*n* = 3), treatment response and survival (*n* = 2), or treatment planning (*n* = 5).

**Conclusion:** Over the past few years, there has been a significant number of studies assessing the performance of DL techniques in radiation oncology. They demonstrate how DL-based systems can aid clinicians in their daily work, be it by reducing the time required for or the variability in segmentation, or by helping to predict treatment outcomes and toxicities. It still remains to be seen when these techniques will be employed in routine clinical practice.

## Introduction

Machine learning (ML) is a vast field that recently gained a lot of interest in medicine (1). Artificial Neural Networks (ANN) are a subfield of ML that mimic the organization of the brain and use several layers of so-called neurons, where each neuron has a weight and a bias that determines its importance. Each layer receives variables, calculates a score and passes the output to the next layer. In radiation oncology, ANNs have been used to predict different outcomes: survival in advanced carcinoma of the head and neck treated with radio(chemo)therapy, PSA-level response and toxicity after radiotherapy for prostate cancer, pneumonitis in radiotherapy for lung cancer, or even survival in uterine cervical cancer treated with irradiation (2–8). The performances of the models created in these studies were satisfying, but the training cohorts were limited, and these models often lacked external validation. In the end, they were never used in clinical routine. Deep Learning (DL) is a new term for ANN arising from advances in the ANN architectures and algorithms since 2006, referring especially to ANN with many hidden layers, although there is no consensus as to how many layers count as deep, therefore there is no clear distinction between the terms ANN and DL [37]. While the interest of DL is being extensively explored in medical imaging for the purposes of classification or segmentation, its use in radiation oncology is still limited (9, 10).

The application of DL techniques to autosegmentation for radiotherapy planning could represent a significant innovation in daily practice workflow, decreasing the time required for segmentation and the variability of the contours and also increasing the adherence to delineation guidelines (11).

Furthermore, real-time adaptive radiotherapy techniques could become a reality with reliable autosegmentation tools based on DL (12).

Level I evidence-based medicine relies on randomized controlled trials designed for specific, pre-defined, populations. However, the high number of parameters that need to be explored to deliver truly individualized cancer care, makes it almost impossible to design trials for every situations (13). New approaches, such as DL used on real-life data, are needed. Data quality is exceptionally high in radiation oncology, as all departments use record-and-verify systems that prospectively store all information regarding the treatment prescribed, how the treatment was actually performed as well as potential deviations. DL could be used to design a “learning health system,” capable of predicting patients' treatment response or survival and constantly updating itself with new data. In this review, we first briefly describe the methods available to create models with DL and then continue with presenting a selection of critical studies published so far that employ DL in radiation oncology. By ordering these studies by entity, and focusing on the clinical relevance, we hope this review will provide a useful resource for clinicians to gain an overview on the current application of deep learning in radiation oncology.

## Methods

The authors conducted a literature review using PubMed/Medline in order to identify important recent publications to be synthesized into a comprehensive review of the current status of Deep Learning in radiation oncology directed at a clinically-oriented reader. The search strategy included the search terms “radiotherapy” and “deep learning.” In addition, reference lists of selected articles were hand searched for further potential hits of relevance to this review. The intervention of interest was the application of deep learning technology in the field of radiation oncology. The search was conducted in April 2018, and mainly identified studies published between 1997 and 2018, strongly skewed toward 2015 and later.

Search results were judged for relevance by the research team using the title, abstract and if necessary full text. Studies were included when they were published as full articles in English employing a deep learning technique in the field of radiation oncology, e.g., for the prediction of side-effects or prognosis.

Studies were excluded for the following reasons: language other than English; no deep learning techniques used; no relevance to the field of radiotherapy; technical investigations without patient data; no clinical application focus or reported outcomes. In the end, 43 studies were included in this analysis. Individual summaries were prepared by a team with a multidisciplinary background (two machine learning scientists and three clinicians) for each study as the basis for further analysis in order to evaluate the technical and clinical aspects. This involved quantitative assessment of the number of patients, frequency and types of techniques used, and comparisons of outcome metrics (if reported). In addition, qualitative assessments were made regarding the reported benefits for radiation oncology practice.

## Deep Learning

### What Is Deep Learning?

The major application areas of deep learning in radiation oncology include image segmentation and detection, image phenotyping and radiomic signature discovery, clinical outcome prediction, image dose quantification, dose-response modeling, radiation adaption, and image generation (2, 3, 10, 14–32). As an additional resource, Meyer et al. provided an excellent review on the technical background, concepts and details of the various different machine learning techniques subsumed under the term deep learning and employed in radiation oncology (33). Of all deep learning techniques, convolutional neural network (CNN) is the most widely used deep learning technique, followed by auto-encoder, deep deconvolution neural network, deep belief network and transfer learning (9, 10, 14, 16, 17, 19, 20, 22, 23, 25, 26, 34–38). CNN, proposed by LeCun et al. for handwritten digit recognition, uses shared “patch” feature detectors across different regions of the input image, dramatically improved learning efficiency (39). Advances in deep learning are quickly applied to radiation oncology as seen by a surge of studies utilizing modern deep learning architectures such as CNN, auto-encoder, and transfer learning since 2017, in contrast to the prevalence of older neural networks such as deep belief networks and fully connected feedforward neural networks in older studies.

Although the application of deep learning in radiation oncology may seem diverse, they share a similar framework. In brief, let *D*:{*X, Y*} denote a dataset containing training examples *X* and their target labels *Y*. In deep learning studies, *X*often represents unstructured data such as CT scans, histology images, in contrast to structured data such as a yes/no tabular referring to whether a patient shows specific symptoms, which is frequently the case in statistical (i.e., non-deep) learning studies. A (supervised) machine learning model, including deep learning, aims to predict *Y* given *X* with a discriminant function *f*:*X* → *Y*^1^. The difference between deep learning and traditional non-deep machine learning methods is that the discriminant function *f* is represented by a neural network. According to the Universal Approximation Theorem, a neural network with a single layer of infinite width can approximate any continuous functions, thus neural networks (namely deep learning) are the most flexible machine learning model family and have the highest model capacity among all machine learning methods (41, 42). To solve for the parameters that determine *f*, a loss function *J*(*f*(*X*), *Y*) is defined and an optimization procedure is applied to determine *f*. One characteristic of deep learning is that most of the useful loss functions are non-convex, in contrast to many machine learning models familiar to the medical community such as support vector machines (SVM) and logistic regression, which means no optimization procedure can guarantee to find the global optimum representing the absolute best choice of *f* (43). This is the reason deep learning is less of an off-the-shelf nature and requires much skill and expertise from the researcher compared to other machine learning techniques. The main advantage of deep learning, however, is its power in analyzing unstructured data, and for extracting useful features (such as edges in images) without any guidance from humans. We continue in this review by highlighting the application of deep learning in image analysis to illustrate the advantages and caveats in utilizing deep learning techniques in radiation oncology.

The input dimension of medical images is usually very high. For example, a pathological image of size 100 by 100 pixels was used in Saltz et al. resulting in 10,000-dimensional input vectors, which can be difficult for non-deep machine learning classifiers, especially when the training samples are not abundant (25). A well-known phenomenon in machine learning research is referred to as the curse of dimensionality, incurring high computational cost for machine learning (40). As a result, dimension reduction is often crucial in medical image classification by machine learning, including deep learning.

Perhaps the best-known dimension reduction technique known outside of the machine learning research community is principal component analysis (PCA) (44). PCA is a linear dimension reduction technique, which transforms the original data by multiplying it with a transformation matrix to achieve minimal covariance among the resulting dimensions, called principal components. However, PCA has a major limitation to its applicability in that it can only model linear interactions. For example, in the sample relation of *y* = *x*_1_ × *x*_2_, where *x*_1_ and *x*_2_ are independent variables, PCA will not be able to distinguish whether *x*_1_ and *x*_2_ are independent influence to *y*, because they are interacting non-linearly.

Deep neural networks can also be used to reduce dimensionality-related issues and untangle non-linear interactions. For example, auto-encoder is a type of ANN that can be used to reconstruct input data by a (often) lower-dimensional hidden layer (45). It is often used in image segmentation and organ detection to learn features automatically (10, 20, 22).

### Deep Learning—Clinical Aspects

The application of deep learning techniques to radiation oncology can offer numerous advantages and significant support to the clinicians in the different steps of radiotherapy treatment, offering the opportunity to both improving technical parameters (i.e., treatment quality and speed) and allowing clinically relevant insights (i.e. survival or toxicity prediction).

**Figure 1:**
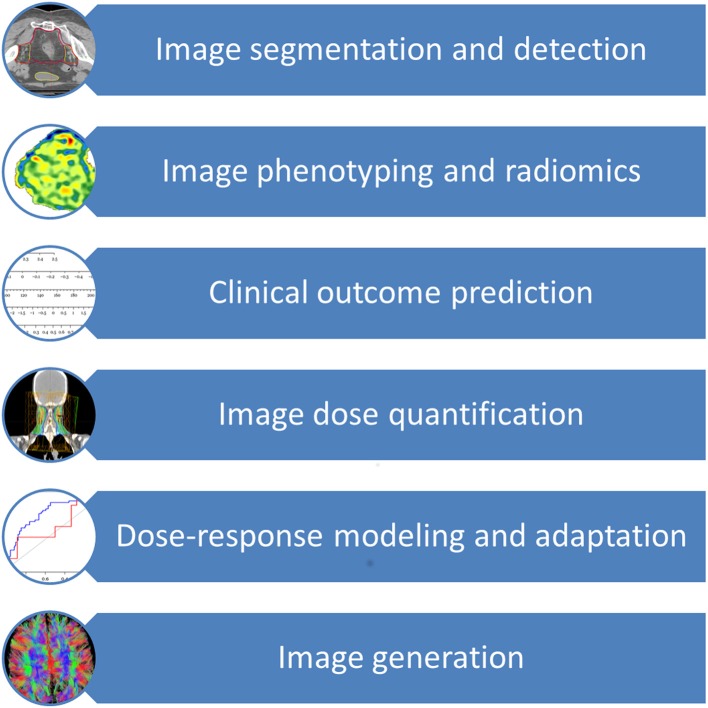
Clinical applications of deep learning in radiation oncology.

The prior knowledge which originated from daily clinical practice can therefore be applied to future patients through these innovative techniques, thereby increasing the quality and effectiveness of their radiation treatment and offering them more tailored approaches. Figure 1 provides an overview of the clinical aspects of DL in radiation oncology. Table 1 summarizes the most significant clinical outcome applications reported in the studies included in this review, whereas Table 2 summarizes the most significant segmentation results.

**Table 1:** Deep Learning for clinical applications in toxicity and outcome prediction as well as treatment planning.

**Outcome**	**Site**	**Deep learning strategy**	**Number of patients/plans**	**Parameter of prediction**	**Measure**	**References**
Toxicity	Prostate	ANN and SVM	321	G2 prediction	ROC 0.7	(4)
Toxicity	Prostate	SVM	256	G2 rectal bleeding	97% prediction accuracy	(25)
Toxicity	Head and Neck	SVM	125	Saliva flow rate	MAPE 1.6%	(25)
Toxicity	Head and Neck	various	47	Hearing loss	AUC 0.7	(42)
Toxicity	Cervix	CNN	42	G2 rectal toxicity	AUC 0.7	(24)
Response	Prostate	ANN	119	Biochemical control	Sensitivity/specificity >55%	(3)
Response	Head and Neck	ANN	95	2-year survival	ROC 0.78	(2)
Planning	Lung	SVM/ANN	9	Real-time gated RT	n/a	(46)
Planning	Lung	ANN	5	Online treatment verification	>97% precision and accuracy	(16)
Planning	Lung	IIFDL	130	Intra- and inter-fractional variation	various	(26)
Planning	Lung	GAN + RAE + DQN	114	Automated dose-adaptation	n/a	(29)
Planning	Pelvis	3D FCN	22	CT image based on MRI	various	(12)

*ANN, Artifical Neural Network; AUC, Area Under the Curve; CNN, Convolutional Neural Network; DQN, Deep Q-Network; FCN, Fully Convolutional Neural Network; GAN, Generative Adversarial Network; IIFDL, Intra- and Inter-fraction Fuzzy Deep Learning; MAPE, Mean Average Percentage Error; RAE, Radiotherapy Artificial Environment; ROC, Receiver Operating Characteristic; RT, Radiotherapy; SVM, Support Vector Machine*.

**Table 2:** Deep Learning for segmentation.

**Site**	**Deep learning strategy**	**Number of patients**	**DICE (reported average or range)**	**References**
Brain	CNN	305	0.67	(32)
	3D CNN	182	0.66	(34)
Head and Neck	DNN	52	0.62 to 0.90	(20)
	CNN	50	0.37 to 0.89	(33)
	DDNN	230	0.33 to 0.81	(36)
Lung	CNN + conditional random fields	30	0.57 to 0.87	(47)
	CNN	450	0.57 (0.16 to 0.99)	(48)
Abdomen	CNN	72	0.7	(17)
	CNN	118	N/A (VOE = 0.06)	(21)
Pelvis	DDNN	230	0.63 to 0.81	(10)
	CNN	140	0.7	(14)
	DDCNN	278	0.62 to 93.4	(36)
	2D CNN	93	0.74	(49)

*CNN, Convolutional Neural Network; DNN, Deep Neural Network; DDN, Deep Deconvolutional Neural Network DICE; DDCNN, Deep Dilated Convolutional Neural Network; VOE, Volumetric Overlap Error*.

#### Segmentation

Deep learning techniques have been widely used in radiology to segment medical imaging obtained through different techniques (46). Segmentation still represents a significant burden in terms of time and human resources in radiation oncology. Artificial intelligence support could help to ensure contouring quality and reduce its intrinsic inter-observer variability as well as the time required for treatment planning.

Auto-segmentation can significantly reduce this burden, with different solutions proposed in the literature as experimental approaches or commercially available software aiding clinicians (11). Several DL techniques have been tested as auto-segmentation tools for radiotherapy purposes.

##### Brain

Numerous experiences have been published about the use of DL techniques in diagnostic neuro-radiology for the segmentation of brain tumors or secondary lesions, but the evidence of its direct use in radiotherapy is still scarce.

Liu et al. recently developed an algorithm to segment brain metastases on contrast-enhanced T1w MRI datasets, with an architecture consisting of four sections: input, convolution, fully connected and classification sections. This approach was validated on data from the Multimodal Brain Tumor Image Segmentation challenge (BRATS−65 patients) and 240 brain metastases patients with T1c MRI scans collected at the University of Texas Southwestern Medical Center.

Segmentation performances were evaluated through Dice Similarity Index (DSI). DSI is a measure for the overlap between two sets of contours (A and B) and is defined as the area of overlap between the contours in exam divided by their mean area:

$$\begin{array}{l} \text{DSI}=\frac{2|\;A\cap B|\;}{\left|A|+|B\right|} \end{array}$$

A DSI of 0 indicates the absence of overlap between the structures, while a value of 1 means that the overlap is complete (50). This study showed DSI values of 0.75 ± 0.07 in the tumor core and 0.81 ± 0.04 in the enhancing tumor for the BRATS data, which outperformed other techniques in the 2015 Brain Tumor Image Segmentation Challenge. Segmentation results of patient cases showed a mean DSI 0.67 ± 0.03 and achieved an area under the receiver operating characteristic curve (AUROC) of 0.98 ± 0.01 (34).

A similar strategy has been proposed by Charron et al. who adapted an existing 3D CNN algorithm (Deep-Medic) to detect and segment brain metastases on MRI scans of patients who were undergoing stereotactic treatments. Authors studied the influence of a combination of multimodal MRIs, the addition of virtual patients in order to increase the total number of patients and the use of advanced segmentation maps to distinguish the necrotic and the vital parts of the metastases. Additionally, in this work, a quantification of the detection performance (defined as the network ability to detect metastases contoured by physicians) has been evaluated through the sensitivity:

$$\begin{array}{l} Sensitivity=\frac{TPmet}{\left(TPmet+FNmet\right)} \end{array}$$

where the TPmet is the number of detected metastases and FNmet the undetected metastases, the measurement of FPmet defined as the number of false-positive lesions and the DSI.

The dataset included 182 patients with three MRI modalities (T1w3D, T2flair2D, and T1w2D) and was split into training, validation and test sets. The benchmark segmentation was carried out manually by up to four radiation oncologists and compared to the DL output. As first step authors set the network parameters to correctly detect the metastases and their segmentation. Six different multimodal MRI configurations have been used (T1w3D, T2flair2D, T1w2D, T1w3D, and T2flair2D, T1w3D, and T1w2D, T1w3D T2flair2D, and T1w2D) as second step to measure the ability of the network for metastases detection and segmentation evaluation. In this study, performance improvement has been shown by using multimodal MRI and in particular using T1w3D plus T2flair2D (Sensitivity = 0.96, FPmet = 9.6 and DSI = 0.77). A slight improvement has been shown using an advanced segmentation map but the addition of virtual patients did not impact significantly on the network.

The obtained results appear to be promising for the application of DL techniques in the identification and segmentation of brain metastases on multimodal MR images (36).

##### Head and neck

Image segmentation of head and neck malignancies is a time-consuming and difficult task in radiotherapy, which could hamper the future developments of adaptive approaches (51). The presence of large primary or nodal lesions and the effects of surgical procedures can significantly modify the normal anatomy of this site, resulting in the need for laborious manual segmentation, as auto-segmentation approaches often fail to manage these anatomical variations. Deep learning techniques and prior knowledge may help in overcoming such difficulties.

CNNs have been used to speed up and improve organs at risk delineation in head and neck cancer patients. A classic CNN structure—repeating blocks of a convolutional layer, a batch normalization layer, a rectified linear unit (“ReLU”) activation layer, a dropout layer, and a pooling layer—was used by Ibragimov and Xing in a tri-planar patch-based network on 50 patients scheduled for head and neck radiotherapy. The CNN's performance was similar or superior when compared to the reference segmentation for the spinal cord, mandible, parotid glands, larynx, pharynx, eye globes and optic nerves, but less satisfactory results have been obtained for the sub-mandibular glands and optic chiasm, mainly due to their dimension and position. One potential reason for the unsatisfactory results for submandibular glands autosegmentation observed by Ibragimov and Xing may be their proximity to oropharyngeal cancers and upper neck positive lymph nodes (35).

Other approaches based on DL and more focused on the delineation of target volumes have also recently been proposed, aiming to speed up and improve the quality of this segmentation process. Men and colleagues developed a deep deconvolutional neural network (DDNN) for the delineation of nasopharyngeal gross tumor volume (GTVnx), metastatic lymph node gross tumor volume (GTVnd), corresponding clinical target volumes (CTVs), and organs at risk in planning CT images of 230 patients diagnosed with nasopharyngeal carcinoma (stage I or II). The results obtained in this study showed an improvement of the consistency in contouring performance and radiotherapy treatment workflow optimization with DL techniques when compared to other segmentation methods (38).

Cardenas and colleagues describe an approach to high risk CTV auto-delineation for head and neck tumors with deep neural networks, demonstrating its potential to reduce the variability in target delineation. Their results are comparable with those reported by inter- and intra-observational studies for manual delineation of these complex volumes (DSI values ranged from 0.62 to 0.90, and the median MSD was 2.75 mm) (22).

##### Lung

Auto-segmentation of the thoracic site with traditional semi-automatic tools showed good performances overall, reaching DSI values above 0.9 when compared to manual benchmark, mainly attributed to the particular anatomy of this site with its naturally high contrast at the air/tissue interfaces (52).

Nevertheless, some interesting DL approaches have been proposed to improve radiotherapy-oriented auto-segmentation performance also in this site, adding to the significant radiological evidence in this field that mainly aims to provide nodule classification and conventional computer-aided diagnosis (CAD) support (9, 46, 53).

Trullo et al. tested an investigational model composed of a 10-layer fully convolutional network with SharpMask, evaluating its segmentation performance against manual definitions of the esophagus, heart, trachea, aorta, and body contour in 30 CT scans of patients affected by lung cancer or Hodgkin lymphoma (54). The best performance was achieved by SharpMask with a conditional random field as input of the deep learning infrastructure to improve the segmentation results providing fine edge details, achieving a 0.67 to 0.9 DSI ratio for different organs (the general DSI ratio was 0.57 to 0.87 depending on the organ) (54).

Lustberg et al. studied the time which can be saved when using software-generated contouring as a starting point for manual segmentation. They tested a commercially available atlas-based software and a prototype of DL software based on CNN for the delineation of thoracic organs at risk (lungs, esophagus, spinal cord, heart and mediastinum) in 20 CT scans of stage I-III lung cancer patients, finding a significant reduction in contouring time with the user-adjusted software-generated contours approach (55).

##### Abdomen

The use of auto-segmentation software, even based on innovative DL techniques, is intrinsically hampered in the abdomen by the significant anatomical variability of this site. Hollow organs, bowel loops displacement and inter-patient variability limit the possibility to use auto-segmentation, generally only achieving relatively poor results. The liver appears to be the ideal candidate for auto-segmentation applications in this anatomical region, due to its usually regular position and shape.

A first attempt for intrahepatic portal vein (PV) segmentation based on CNN has been proposed by Ibragimov et al. as support for stereotactic body radiation therapy (SBRT) planning (19). The portal vein is usually poorly visible in planning images (e.g., due to artifacts, fiducial, stents, variable anatomy) and its segmentation is often challenging even for expert operators. However, automated DL-based segmentation algorithms could satisfactorily support segmentation with DSI of 0.7–0.83 when compared to manual benchmarks. Results on a par with manual delineation were also found by Lu and colleagues, who developed and validated a CNN with a graphcut-based strategy for liver auto-segmentation using 78 CT as training set and 40 as test set (23).

##### Pelvis

Several automatic strategies for pelvic auto-segmentation have been assessed in the recent years, and the development of more efficient DL technique may improve the current state-of-the-art (56, 57).

Deep learning applications have been recently used as segmentation support in the pelvic region, for both organs at risk and MRI-based segmentation of target volumes. Men et al. used a deep dilated convolutional neural network-based method to automatically segment organs art risk and CTVs in planning CTs of rectal cancer patients, obtaining a 87.7% concordance for target volumes with very high segmentation speed (45 s) (38).

Trebeschi and colleagues proposed a 9 layers CNN method for primary locally advanced rectal cancer lesions on T2 and DWI MRI images, obtaining a DSI of 0.7 and a model's area under the curve (AUC) of 0.99 in the comparison of CNN-generated vs. manually obtained contours (16).

Similar results were also reached by Wang et al. who implemented a 2D CNN auto-segmentation model for GTV segmentation on T2 MR images of 93 rectal cancer patients, showing a segmentation performance comparable to manual inter-observer variability (DSI 0.74) (47). An interesting DL application for MRI for prostate cancer has been developed by Guo et al. proposing to learn the latent feature representation from prostate MR images using a stacked sparse auto-encoder (SSAE), which included an unsupervised pre-training step and also a task-related fine-tuning step on 66 patients (10).

#### Outcome Prediction

##### Toxicity

A promising field to apply DL techniques in radiation oncology is predicting toxicity as a valuable decision support system for the clinician (48). A first machine learning study has been published in 2009 by Zhang et al. describing a plan-related clinical complications prediction model in an IMRT framework. one hundred and twenty-five plans were generated for one head and neck cancer case and another 256 plans were generated for one prostate cancer case, with saliva flow rate and G2 rectal bleeding being used as prediction outcomes. The mean absolute error for saliva flow rate prediction compared with the ground truth obtained from the equivalent uniform dose (EUD) exponential model was 0.42%, while an average prediction accuracy of 97.04% was achieved for G2 rectal bleeding. A direct inference of plan-related complications using the knowledge-base of computed plans and modeling as described in this work therefore appears to be feasible and promising for further DL applications (27).

Pella and colleagues recorded clinical and dosimetric data of 321 prostate cancer patients, scoring gastro-intestinal and genito-urinary acute toxicities and stratifying patients into a mild and a severe toxicity category (with a cut-off of G2 acute toxicity according to Radiation Therapy Oncology Group (ROG)/European Organization for Research and Treatment of Cancer (EORTC) scale); the resulting neural networks and SVM-based solutions showed comparable toxicity prediction accuracy, exhibiting a AUC of 0.7 (4).

Rectal toxicity prediction in cervical cancer radiotherapy has been studied by Zhen and colleagues developing a CNN model based on transfer learning that analyzed the dose distribution in the rectum in 42 patients and predicted the relative > G2 rectal toxicity with an AUC of 0.7 (26).

More recently, Adbollahi et al. conducted a multi-variable modeling study on sensorineural hearing loss in head and neck cancer patients treated with radiochemotherapy using CT radiomics information: the average obtained predictive power of the tested methods was higher than 70% in accuracy, precision and AUROC (58).

##### Response and survival

The use of DL techniques for response and survival prediction in radiotherapy patients represents a great opportunity to further develop decision support systems and provide an objective evaluation of the relative benefits of various treatment options for individual patients.

Bryce et al. analyzed data from a phase III trial randomizing 95 patients with locally advanced squamous cell carcinoma of the head and neck (SCCHN) to undergo irradiation with or without concurrent chemotherapy. Prediction power was evaluated using the round-robin cross-validation method and ROC analysis. The best ANN model used tumor stage, nodal stage, tumor size, tumor resectability, and hemoglobin to predict 2-year survival with an area under the ROC curve value of 0.78 ± 0.05, confirming its ability to identify and use predictive variables other than the TNM variables and to classify each patient individually (2).

A more recent model has been explored to predict biological outcomes for patients receiving radical radiotherapy for prostate cancer, using a three-layered ANN (Perceptron) model on retrospective clinical data, learning the relationship between the treatment dose prescription, dose distribution and the corresponding biological effect in 119 patients.

In this study, the ANN was able to predict biochemical control and specific bladder and rectum complications with sensitivity and specificity of above 55% when the outcomes were dichotomized (3).

##### End-to-end deep learning radiomics pipeline

We are currently witnessing a turning point in the radiomics methodologies used in treatment response prediction and prognosis. Most traditional radiomics methods use handcrafted features and involve manual segmentation of the region of interest (e.g., the tumor) on medical imaging, and extraction of thousands of hand-crafted and curated quantitative features from the region of interest, which supposedly describe tumor characteristics. This could actually introduce human bias into the process, raising concerns of reproducibility (49, 58, 59). The intra-reader and inter-reader variability that results from manual segmentation of the tumor and the variation in imaging and pre-processing techniques for feature extraction could significantly impact the models that are created afterwards. Deep learning allows for automated segmentation, extraction and learning of relevant radiographic features without the need for human intervention in the analysis pipeline. For that reason, DL could boost reproducibility, generalizability and accuracy and reduce potential bias (60).

##### Planning

Over the past years, a growing role of DL techniques in radiotherapy planning optimization has been observed, especially for lung cancer.

Lin et al. proposed a model for real time detection of lung cancer targets in a treatment gating window using a sequence of 10 fluoroscopic images of nine patients. Different combinations of dimension reduction techniques and DL based classifiers (SVM and ANN) were tested. The ANN combined with principal component analysis (PCA) approach appeared to be a more effective algorithm compared to the other combinations for real time gated radiotherapy, despite the study's conclusions being drawn using the same patient's image sequence as training and testing data (61).

A similar study was conducted by Tang and colleagues, who proposed an ANN-based algorithm for online treatment verification by monitoring tumor position in 5 lung cancer patients undergoing hypofractionated radiotherapy, using digitally reconstructed radiographs (DRR) with artificially added shifts in tumor location and PCA for dimension reduction from 10,000 from 100 by 100 cine images to 15 top principal components for classification. An accuracy of 98.0%, recall of 97.6% and precision of 99.7% were observed (18).

The prediction of intra- and inter-fractional variations of lung tumors has been further investigated by Park and colleagues based on the breathing data of 130 patients. The proposed model is based on fuzzy DL technique and was compared with CNN. The proposed model improved root-mean-square-error by 29.98% and prediction overshoot by 70.93% compared with the other existing methods. The average computing time for intra- and inter-fraction fuzzy deep learning (IIFDL) was 1.54 ms for both intra- and inter-fractional variations, which is smaller than existing methods, confirming the advantages in the use of this technique (28).

An innovative delivery optimization technique for automated plan adaptation in lung cancer radiotherapy based on deep reinforcement learning has been proposed by Tseng et al. with the aim to maximize tumor local control and reduce rates of radiation-induced Grade 2 pneumonitis.

A retrospective cohort of 114 NSCLC patients receiving radiotherapy was used as training set. Input features included clinical, genetic and radiomics information in addition to tumor and lung dosimetric variables. A validation cohort of 34 patients treated on avid PET signal in the tumor with real clinical protocols was used as test set for benchmarking.

The proposed model generated dose distributions similar to the clinical protocol ones, with a root mean square error of 0.5 Gy; at the same time, the neural network-based decisions seemed to have a superior concordance with patients' eventual outcomes. The authors concluded that deep reinforcement learning is a feasible and promising approach for automatic radiation adaptation, that currently represents a intensively studied radiation oncology delivery technique (31).

An innovative approach for simulation workflows has been proposed by Nie et al. who developed 3D fully convolutional networks to estimate CT imaging from MRI data based on a pelvic dataset of 22 patients. When compared to the classical approach (atlas-based methods), 3D convolutional neural network (CNN) was able to capture the complex non-linear mapping from the input space to the output space and generate a structured output which can better preserve the neighborhood information in the predicted CT image.

The most immediate clinical advantage of this approach is that it could avoid patients to undergo the simulation CT, reducing unnecessary X-ray exposure This clearly is a very contemporary topic considering the emergence of innovative applications such as MR guided hybrid radiotherapy (MRgRT) (14, 62).

## Discussion and Conclusion

This review presents an overview on the application of deep learning techniques in radiation oncology. Synthesizing a total of 43 studies, it can be concluded that over the past few years there has been a significant increase in both the interest in as well as the performance of DL techniques in this field.

Promising results have been obtained that demonstrate how DL-based systems can aid clinicians in their daily work, be it by reducing the time required for or the variability in segmentation, or by helping to predict treatment outcomes and toxicities. It remains to be seen when these techniques will be employed in routine clinical practice, but it seems warranted to assume that we will see AI to contribute to improving radiotherapy in the near future.

We found during this review that it is common in radiation oncology literature to use deep learning only for dimension reduction or feature extraction, then employing a separate non-deep classifier, such as SVM or logistic regression, to perform the classification on extracted features (20). The advantage of this approach is its straightforward framework and the fact that the extracted features are accessible for additional analyses such as risk evaluation by studying their associations with disease phenotypes (63). Although this approach does yield better performance than using machine learning classifiers on handcrafted features, end-to-end deep learning approach usually perform better if properly trained (43). In oncology studies, the number of human subjects is usually a limiting factor for the statistical power of the findings of the study; therefore, the previous approach will be highly restricted by the number of human subjects recruited or by the number of medical images obtained for a study, while end-to-end approaches can leverage powerful pre-trained model such as Google Inception (64). These models are trained on millions of images, and the researchers only need to fine tune the last layer to refine the decision boundary for medical image analysis. In addition, the hidden layers are more likely to extract agnostic features which may lead to the discovery of new biomarkers (24).

Another caveat is that most studies so far use relatively small patient cohorts or numbers of training images. For example, only 52 CT scans were used in the image segmentation study by Cardenas et al. and only 42 patients were included in the radiation toxicity prediction reported by Zhen et al. (22, 26). To train a deep neural network effectively, a rule of thumb is to use 5,000 labeled examples as training input (43).

Neural networks have a relatively high model capacity compared to other machine learning techniques such as SVM or logistic regression. This means that they are prone to over-fitting small datasets, and therefore special caution needs to be applied when making comparisons of neural network and other machine learning techniques on the classification of small datasets. One solution is to validate on a similar dataset obtained from a different cohort (20). Another solution is to build large imaging datasets such as those in the UK Biobank (http://www.ukbiobank.ac.uk/imaging-data/).

In conclusion, the application of deep learning has great potential in radiation oncology. However, it is less of an “off-the-shelf” in nature than other machine learning techniques such as SVM, ensembles, k-nearest neighbors algorithm (KNN), and logistic regression, as it has more hyper-parameters to tune and requires more technical skills from the researchers. Deep learning continues to be an important research area and many aspects of training neural networks remain “more of an art than science” (43).

Finally, we would also like to highlight the need to make bigger standardized datasets available for future collaborative endeavors. This would hopefully enable researchers to develop more robust algorithms for clinical use, thereby facilitating the introduction of new technologies into daily care, ultimately leading to improved outcomes for cancer patients.

## Author Contributions

All authors contributed equally in the literature research, writing, and editing of the manuscript.

### Conflict of Interest

The authors declare that the research was conducted in the absence of any commercial or financial relationships that could be construed as a potential conflict of interest.

## Footnotes

^1^Readers more familiar with machine learning may know this is only one of the three major machine learning perspectives, with the other two being generative modeling and discriminative modeling (40). Without delving too deep into the technical details of machine learning, we explain the principles of machine learning specifically from this intuitive perspective.
